# Polycaprolactone/Polyethylene Glycol/Hydroxypropylmethylcellulose Blends: Tailoring Thermomechanical and Rheological Properties for Injection-Molded Capsules for Colon-Targeted Delivery Applications

**DOI:** 10.1021/acsabm.5c01503

**Published:** 2025-12-08

**Authors:** Stefania Mottola, Sara Liparoti, Andrea Miranda, Iolanda De Marco

**Affiliations:** Department of Industrial Engineering (DIIn), 19028 University of Salerno, Via Giovanni Paolo II, 132, Fisciano, SA 84084, Italy

**Keywords:** HPMC, injection molding, thermomechanical properties, release profiles, pH-sensitive polymer blends, colon-targeted drug delivery

## Abstract

Colon-targeted delivery systems offer a promising approach for local drug administration. In this study, we developed a customized polymeric blend for this purpose, combining polyethylene glycol (PEG), polycaprolactone (PCL), and hydroxypropyl methylcellulose (HPMC). Although PEG and PCL have been extensively studied, the inclusion of HPMC in such blends remains underexplored; however, its use in this context shows significant potential due to its pH sensitivity. To achieve this, various formulations were tested to optimize the thermomechanical and release characteristics of capsules produced through injection molding. Three blends containing 22, 24, and 34 wt% HPMC were processed and analyzed using rheological methods, ATR-FTIR, TGA, DSC, SEM, and in vitro release tests with methylene blue as a model compound. Simulated pH-release tests (pH 2.5, 5, and 6.8) showed minimal release in gastric and intestinal environments, with controlled and sustained release under colonic pH conditions. It was also observed that the initial HPMC content affects the release rate of the model compound. Specifically, when the blend contains 34% HPMC, approximately 38% of the compound is released within 12 h and 73% within 24 h. These results highlight the potential of pH-sensitive polymer blends as effective platforms for colon-targeted drug delivery. A model illustrating how the release rate depends on pH value and HPMC amount was also proposed and validated. The process was considered to happen in two stages: initially, the release medium penetrates the capsule and solubilizes the model compound; then, the model compound is released into the surrounding environment.

## Graphical abstract

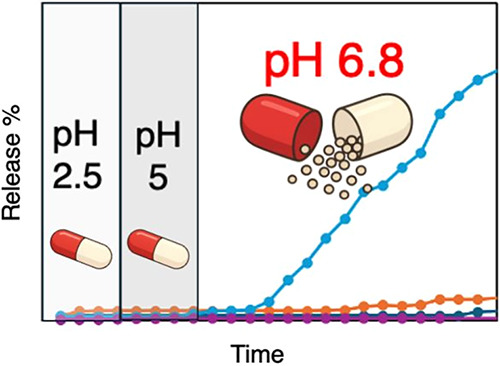

## 1 Introduction

Colon-targeted drug delivery systems are specialized devices designed to transport active compounds directly to the colon. This targeted approach offers notable therapeutic advantages, including enhanced treatment efficacy, reduced systemic side effects, and improved patient adherence to therapy.^1,2^

Conventional oral drug delivery methods often present limitations, such as premature drug degradation before the compound reaches the colon.^2^ To address these challenges, controlled and targeted delivery systems employ various strategies, including time-dependent release mechanisms and polymeric formulations that respond to pH variations.^3^

Directly targeting the colon enhances the treatment of local diseases by increasing drug concentration at the site of action and reducing systemic exposure and toxicity. This strategy can also improve drug bioavailability.^4,5^ Advances in polymer science and nanotechnology have further enabled the precise control of drug release profiles, ensuring that therapeutic agents remain stable until reaching their intended target.^6^

Additionally, the scope of colon-targeted systems has expanded beyond local therapies, now encompassing systemic delivery of sensitive compounds such as proteins, peptides, and vaccines, which are vulnerable to degradation in the upper gastrointestinal tract.^7^

As research continues to evolve, colon-targeted delivery systems show consistent potential to improve treatment outcomes and promote patient-focused therapeutic approaches.^8,9^

Colon-specific formulations include both oral and rectal routes. The first oral delivery system targeting the terminal ileum and proximal colon, known as ileocolonic delivery, was introduced in 1982. It employed a pH-sensitive methacrylate polymer Eudragit S (Evonik),^10^ as an enteric coating. This polymer enables drug release in the terminal ileum and remains widely used in commercial products. However, clinical studies have shown variability in release behavior, with coatings occasionally dissolving prematurely or failing to release the full content in some individuals.^11^

In this context, one of the main challenges is developing specific polymeric blends, which are a promising strategy for creating advanced drug delivery systems, especially those that require targeted and controlled release.^12^

By combining two or more polymers with complementary properties, the physical, chemical, and biological characteristics of the final formulation can be customized.^13^ Choosing the right polymers is essential to attain the desired drug release profile, mechanical strength, biocompatibility, and therapeutic objectives. Numerous studies in the literature have suggested blending different materials to develop tailored materials with specific properties and applications.^14–16^

Both natural and synthetic polymers offer distinct advantages, and their combination may overcome individual limitations.^17,18^

One such polymer is Polycaprolactone (PCL), a biodegradable polymer known for its high biocompatibility and slow degradation rate.^19^ These characteristics make it ideal for long-term and sustained drug release systems. Due to its hydrophobicity, PCL also efficiently encapsulates lipophilic drugs.^20^ When blended with other polymers, PCL contributes to structural stability and facilitates controlled drug release, making it highly suitable for colon-targeted delivery and other biomedical applications.^21–23^

Another relevant polymer is Polyethylene glycol (PEG), widely used for its plasticizing properties.^24^ PEG enhances flexibility and processability by reducing intermolecular forces within the polymer matrix. It is nontoxic, water-soluble, biodegradable, and environmentally friendly – qualities that make it suitable for both biomedical and packaging applications. Its plasticizing action improves polymer processability and mechanical resilience.^25^

Hydroxypropylmethylcellulose (HPMC) is another polymer suitable for targeted drug delivery, due to its pH-responsive behavior.^26^ HPMC is a nonionic compound whose solubility and swelling properties vary with pH, enabling controlled drug release in the gastrointestinal tract. It remains stable in acidic environments, preventing premature drug release in the stomach, and swells significantly in neutral to basic pH, thereby facilitating drug diffusion.^27^ These properties make HPMC highly suitable for sustained and site-specific delivery systems.^28^

Among the available techniques for producing controlled-release systems from polymeric blends, injection molding is one of the most promising for capsule manufacturing.^29–31^

Building on these insights, the present study aims to optimize a polymeric blend for colon-targeted drug delivery by developing a release profile aligned with gastrointestinal transit time, using the injection molding technique. Different formulations were prepared by varying the ratios of PCL, PEG, and HPMC to investigate their effects on active compound release timing and mechanical characteristics. Methylene blue was used as a model compound to assess the release profiles of the blends.

Many studies in the literature discuss the use of individual polymers in colon-targeted delivery systems, but there is limited research on optimizing polymer blends and producing capsules with this approach. The optimization of the properties of these customized blends, and the release behavior from the final capsules, can be interesting for the delivery of some specific drugs in diseases that affect the gastrointestinal apparatus.

## 2 Materials and Methods

### 2.1 Materials

Polycaprolactone (M_n_ = 80 000 uma; *M*_w_/*M*_n_ < 2), and Polyethylene glycol (M_n_ = 15 000 uma), and Hydroxypropyl cellulose (20–60 mPa s) were purchased from Sigma-Aldrich (now Merck KGaA, Saint Luis). PCL and PEG were used after a drying procedure conducted under vacuum at 30 °C for 2 h. HPMC was dried under vacuum at 80 °C for 24 h. Methylene was purchased from Thermo Fischer Scientific (Waltham).

### 2.2 Extrusion and Injection Molding of the Blends

A microcompounder (HAAKE MiniLab II Micro Compounder, by Thermo Scientific) with an integrated backflow channel was adopted to prepare the PCL/PEG/HPMC blends. The materials were mixed at 120 °C and 5 rpm, and a backflow time of 4 min was adopted. Several PCL/PEG/HPMC ratios were adopted, as listed in Table 1.

**Table 1:** PCL/PEG/HPMC Percentages Adopted for the Production of the Blends

	blend	percentages (on total weight of polymers)
1	PCL/PEG	80/20
2	PCL/PEG/HPMC	65/20/15
3	PCL/PEG/HPMC	55/20/25
4	PCL/PEG/HPMC	45/20/35

Injection molding tests were conducted by a mini-injection molding machine, Haake MiniJet II (Thermo Scientific). This apparatus consists of a pneumatic piston that pushes the material from the hot cylinder to the mold. The mold was kept at 80 °C to favor the formation of strong weld lines. The piston was maintained at 120 °C, and the melting process lasted 5 min. An injection pressure of 50 bar was applied. The capsule was extracted from the mold after it had cooled down to room temperature.

### 2.3 Analytical Techniques

#### 2.3.1 Attenuated Total Reflectance/Fourier Transform Infrared Spectroscopy (ATR-FTIR)

Fourier-transform infrared (FTIR) spectroscopy was conducted on the polymer blends using a PerkinElmer instrument (Spectrum 100, PerkinElmer Holdings Ltd., UK) in the range 4000–650 cm^–1^, with a resolution of 4 cm^–1^, in ATR mode. To identify the characteristic peaks of PCL and PEG, the ranges 1790–1650 cm^–1^ and 1150–1070 cm^–1^ were analyzed in detail. Each spectrum has been normalized to the peak at 1470 cm^–1^.

#### 2.3.2 Thermogravimetric Analysis (TGA)

TGA analyses were performed using a TGA/DSC 3+ Mettler Toledo (Columbus) instrument. The employed protocol involves a first heating step from 25 to 700 °C under a 50 mL/min nitrogen flow, followed by a second heating step from 700 to 1000 °C under a 100 mL/min air flow. For both heating steps, the heating rate was kept constant at 10 °C/min.

#### 2.3.3 Differential Scanning Calorimetry (DSC)

The PCL and PEG melting temperatures, as well as the adequate amount of HPMC in the blends, were determined using a DSC 3+ Mettler Toledo (Columbus) instrument.

The following protocol, consisting of four steps, has been used:

- the sample was heated from 0 to 200 °C at 10 °C/min;
- the sample was maintained at 200 °C for 5 min;
- the sample was cooled from 200 to 0 °C at 1 °C/min;
- the sample was heated from 0 to 200 °C at 3 °C/min.

The first heating step is necessary to erase the thermomechanical history that the sample underwent during extrusion. Then, after an isothermal phase, a slow cooling is required to allow the PCL and PEG crystallization.

It is important to note that HPMC is an amorphous polymer and, therefore, it does not have a melting peak, unlike PCL and PEG, which show two separate melting peaks. The percentage of PCL (or PEG) was calculated by comparing the melting enthalpy of the second heating step with that of the pure polymer

1 $${\%}_{polymer\;in\;the\;blend}=\frac{{\Delta H}_{m,polymer\;in\;the\;blend}}{{\Delta H}_{m,pure\;polymer}}100$$

The percentage of HPMC in the blend represents the complement to the summation of PCL and PEG percentages

2 $${\%}_{\mathrm{HPMC}}=100-{\%}_{\mathrm{PCL}}-{\%}_{\mathrm{PEG}}$$

#### 2.3.4 Rheology

Rheological measurements were performed to evaluate the effect of HPMC on the viscoelastic properties (complex viscosity η*, storage modulus G′, and loss modulus G″) of PCL/PEG blends. A stress-controlled rotational plate–plate rheometer, Thermo Scientific HAAKE MARS 60, was used to perform the measurement. A stress sweep test conducted between 10 and 1000 Pa allowed for the identification of the region exhibiting linear viscoelastic behavior. Frequency Sweep tests were performed at 100, 120, and 140 °C with a stress value τ of 100 Pa and a frequency range of 0.1 to 100 rad/s.

#### 2.3.5 Dynamic Mechanical Analysis

Dynamical mechanical analyses were performed using a DMA 8000 system (PerkinElmer, Waltham). This type of analysis is essential for understanding how materials respond to cyclic stresses and temperature variations. Rectangular samples were obtained from the extruded blends. These were tested in temperature-scan mode over the range 20–50 °C. The heating rate was 2 °C/min, with a frequency of 15 Hz (identified following a frequency sweep test).

#### 2.3.6 Scanning Electron Microscopy (SEM)

A scanning electron microscope (SEM) (Phenom ProX, Phenom-World BV, Eindhoven, The Netherlands) was used for the morphological characterization. A sputter coating of a conductive material (gold) is applied onto the samples to avoid charge buildup on the surface. A layer of 10 nm was sputtered onto the specimen surface.

#### 2.3.7 UV/vis Spectrophotometry

Release profiles were evaluated using a Varian Cary 50 UV/vis spectrophotometer (Palo Alto, CA) at a wavelength of 665 nm. Capsules containing 3 mg of methylene blue (MB) were placed in a paper filter (50 μm average porosity) and then immersed in 300 mL of the release medium; the system was continuously stirred at 150 rpm and 37 °C. Given the goal of this work to produce capsules for colon-targeted release, the tests were carried out to simulate the transit of the gastrointestinal tract. As a result, capsules containing MB were placed in contact with different release media, i.e., 1 M hydrochloric acid solution at pH = 2.5 (to simulate transit in the stomach), phosphate-buffered saline (PBS) at pH = 5 (to simulate transit in the small intestine), and PBS at pH = 6.8 (to simulate transit in the colon).

Three calibration curves were determined using diluted standards at five different MB concentrations in each release medium; the curves were then used to convert absorbances (*y*) to MB concentrations (x in mg/mL). The calculated calibration curves had the form:

- *y* = 164.74 x (*R* ^2^ = 0.9971) for the release medium at pH = 2.5
- *y* = 163.57 x (*R* ^2^ = 0.9987) for the release medium at pH = 5
- *y* = 174.28 x (*R* ^2^ = 0.9906) for the release medium at pH = 6.8.

The release tests were performed according to the following protocol: the sample was put in contact for 1.5 h with the medium at pH = 2.5, then it was placed in PBS at pH = 5 for 2 h, and, for the time necessary to reach the plateau, it was put in contact with PBS at pH = 6.8.

## 3 Results and Discussion

### 3.1 Characterization of the Polymer Blends

#### 3.1.1 ATR-FTIR of Pure Polymers and Blends

Figure 1a shows the ATR-FTIR spectra of the neat PCL, PEG, and HPMC materials. It is possible to observe characteristic peaks in the PCL spectrum, such as 2927 cm^–1^ (asymmetric −CH_2_ stretching), 2840 cm^–1^ (symmetric CH_2_ stretching), and 1726 cm^–1^ (C=O stretching).^32^ PEG spectrum shows some characteristic peaks at 843 cm^–1^ (C–O stretching, C–C stretching and CH_2_ rocking), 947 cm^–1^ (CH_2_ rocking and CH_2_ twisting), 1097 cm^–1^ (C–O and C–C stretching), 1280 cm^–1^ (CH_2_ twisting) and 2850 cm^–1^ (CH_2_ symmetric stretching).^33^ HPMC sample shows absorbance peaks at 3415 cm^–1^ (OH stretching vibration), 2927 and 2858 cm^–1^ (C–H stretching vibration), 1634 cm^–1^ (water in the amorphous region), 1055 cm^–1^ (C–O–C stretching).^34^

**Figure 1:**
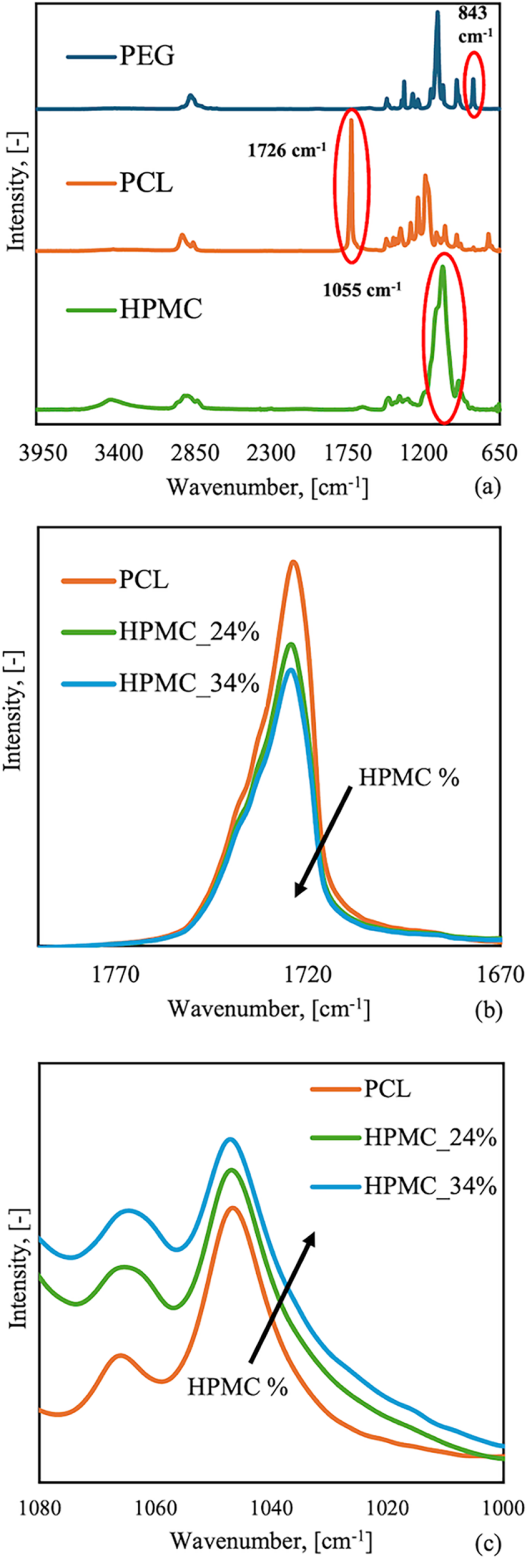
(a) ATR-FTIR traces with the characteristic peaks of each material. (The red circle in the figure indicates the distinctive peak of each material, which is 1726 cm^–1^ for PCL, 843 cm^–1^ for PEG, and 1055 cm^–1^ for HPMC.) (b) ATR-FTIR spectra of pure PCL and the blends containing different percentages of HPMC. Enlargement of the 1790–1670 cm^–1^ region, detail of the characteristic PCL peak. (c) ATR-FTIR spectra of pure PCL and the blends containing different percentages of HPMC. Enlargement of the 1080–1000 cm^–1^ region, detail of the characteristic HMPC peak.

Three characteristic peaks have been identified in Figure 1a, for PCL, PEG and HPMC, respectively: the chosen peaks for each material are 1726 cm^–1^ for PCL, 843 cm^–1^ for PEG, and 1055 cm^–1^ for HPMC.

Figure 1b highlights the effect of HPMC on the characteristic peak of PCL. A comparison of the spectra at 1726 cm^–1^ shows that as the HPMC amount increases, the intensity of this peak decreases (see Figure 1a). Figure 1c shows the HPMC characteristic peak at 1055 cm^–1^ increasing in intensity as the percentage of HPMC in the blend increases. Although ATR-FTIR is not a quantitative technique, the analysis confirms the presence of HPMC in each blend at levels consistent with the theoretical loading.

#### 3.1.2 TGA of the Pure Polymers and Blends

Thermo-oxidative degradation of pure polymers and polymeric blends was assessed using TGA analysis (with the thermograms provided in the Supporting Information). The TGA curve for pure HPMC, shown in Figure S1, indicates thermal degradation occurring within the range of 310–389 °C.^35^ In contrast, both PCL and PEG degraded at higher temperatures (in the range 380–450 °C). As shown in Figure S2.1, the thermal degradation of the blends occurred at progressively lower temperatures with increasing HPMC content. This trend is further confirmed by the first derivative of the TGA curves (DTG), reported in Figure S2.2.

#### 3.1.3 DSC of the Pure Polymers and Blends

Calorimetric analyses were conducted following the protocol described in Section 2.3.3. The thermograms corresponding to the second heating step of the pure polymers are reported in Figure S3. It is evident that HPMC is an amorphous polymer and does not present any melting peak, whereas PCL and PEG show melting temperatures at 57 and 67 °C, respectively. DSC analyses enable the calculation of HPMC content in the blends according to eqs 1, 2. The melting enthalpies were calculated by measuring the areas of the endothermic peaks. Figure 2 shows the thermograms of the blends obtained during the second heating step (thermograms of PCL and PEG are also reported for comparison).

**Figure 2:**
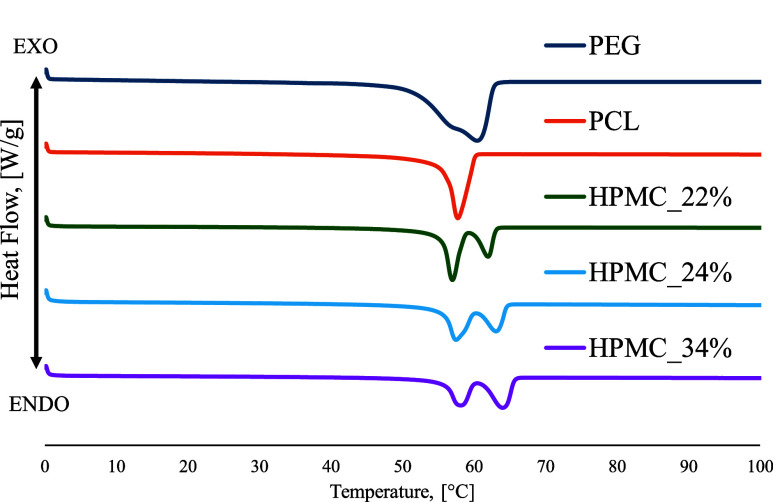
Calorimetric analyses conducted on neat materials and blends.

Blends are characterized by two melting peaks at 57 °C (attributable to PCL) and 62 °C (attributable to PEG). The melting enthalpies are equal to 63.22 J/g and 121.13 J/g, for PCL and PEG, respectively. The effective percentages of the polymers are reported in Table 2.

**Table 2:** Melting Enthalpies of PCL and PEG in the Blends and HPMC Real Percentages

PCL/PEG/HPMC theoretical percentage	|Δ*H*_PCL_blend__| [J/g]	|Δ*H*_PEG_blend__| [J/g]	PCL/PEG/HPMC real percentage
65/20/15	41.24	16.01	65/13/22
55/20/25	37.15	20.15	59/17/24
45/20/35	28.94	24.68	46/20/34

The real percentages differ from the theoretical ones. This finding is consistent with expectations, as melt compounding shows low mixing efficiency.

#### 3.1.4 Rheological Analyses

Rheological tests were performed to evaluate the effect of HPMC addition on the behavior of PCL/PEG blends. Measurements were conducted over a temperature range of 80–140 °C. For each blend the viscosity was measured at different temperatures (see Figure S4) in order to derive the temperature-dependent behavior. The shift factor α_T_, which describes such temperature dependence of viscosity, was subsequently calculated. The resulting shift factors for the various blends are listed in Table 3.

**Table 3:** Shift Factor α_T_ for Different Blends; α_T_ = 1 for a Temperature of 100 °C

blend	*T*	α_T_
PCL/PEG	80	1
	90	0.77
	100	0,61
59/17/24	100	1.36
	120	1
	140	0.82
46/20/34	100	1,40
	120	1
	140	0.89

Single curves were shifted at the same temperature of 120 °C according to factor α_T_ to obtain master curves for each blend. Figure 3 shows the master curves at a reference temperature of 120 °C for the three polymeric blends. This temperature was selected based on the conditions used during the injection molding process. The curves clearly demonstrate that increasing the HPMC content leads to an increase in viscosity.

**Figure 3:**
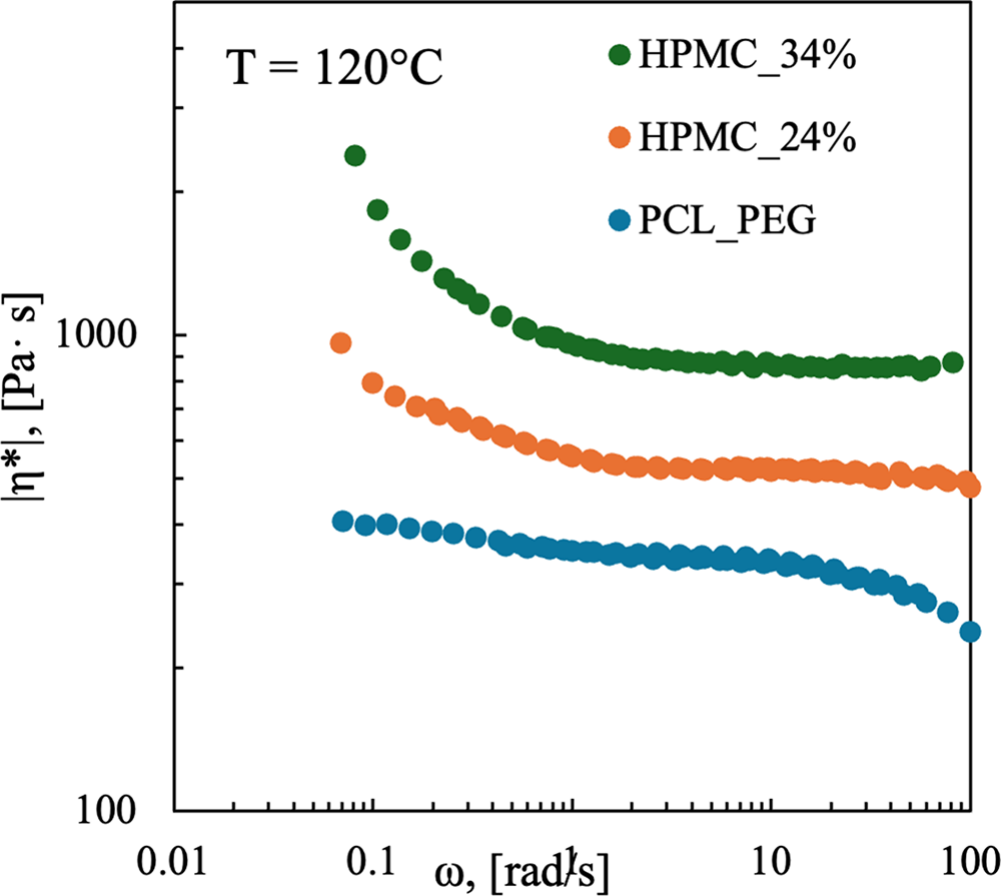
Complex viscosity of the PCL/PEG and PCL/PEG/HPMC blends. The master curves are referred to a temperature of 120 °C.

For the PCL/PEG blend, the viscosity remains nearly constant within the analyzed frequency range. No power-law dependence of viscosity on frequency (shear rate) is observed, and the viscosity values are relatively low, approximately 200 Pa·s. This finding is consistent, as the selected temperature is significantly higher (by over 70 °C) than the melting temperature of the polymers, resulting in Newtonian behavior.

In contrast, HPMC behaves more like a solid filler. Thus, as the HPMC concentration increases, the blend viscosity increases, and at low frequencies the material exhibits a Herschel-Bulkley behavior. In highly filled polymer systems, the viscosity increases at low frequencies, and a yield point may appear. This indicates that a minimum applied stress is required to initiate flow. This phenomenon is caused by interactions among the filler particles and between the fillers and the polymer matrix.^36^

#### 3.1.5 Dynamic Mechanical Analysis (DMA)

Dynamic Mechanical Analysis in tension was performed on the obtained blends. An increase in the HPMC content resulted in a corresponding rise in the storage modulus, indicating an enhancement in the system’s rigidity.^37^ The HPMC content was found to moderately influence the thermal response of the blends, as indicated by an earlier deflection in the storage modulus shown in Figure 4. However, the modulus’ decrease at higher temperatures is not a concern, as these temperatures are not compatible with the physiological human body temperature.

**Figure 4:**
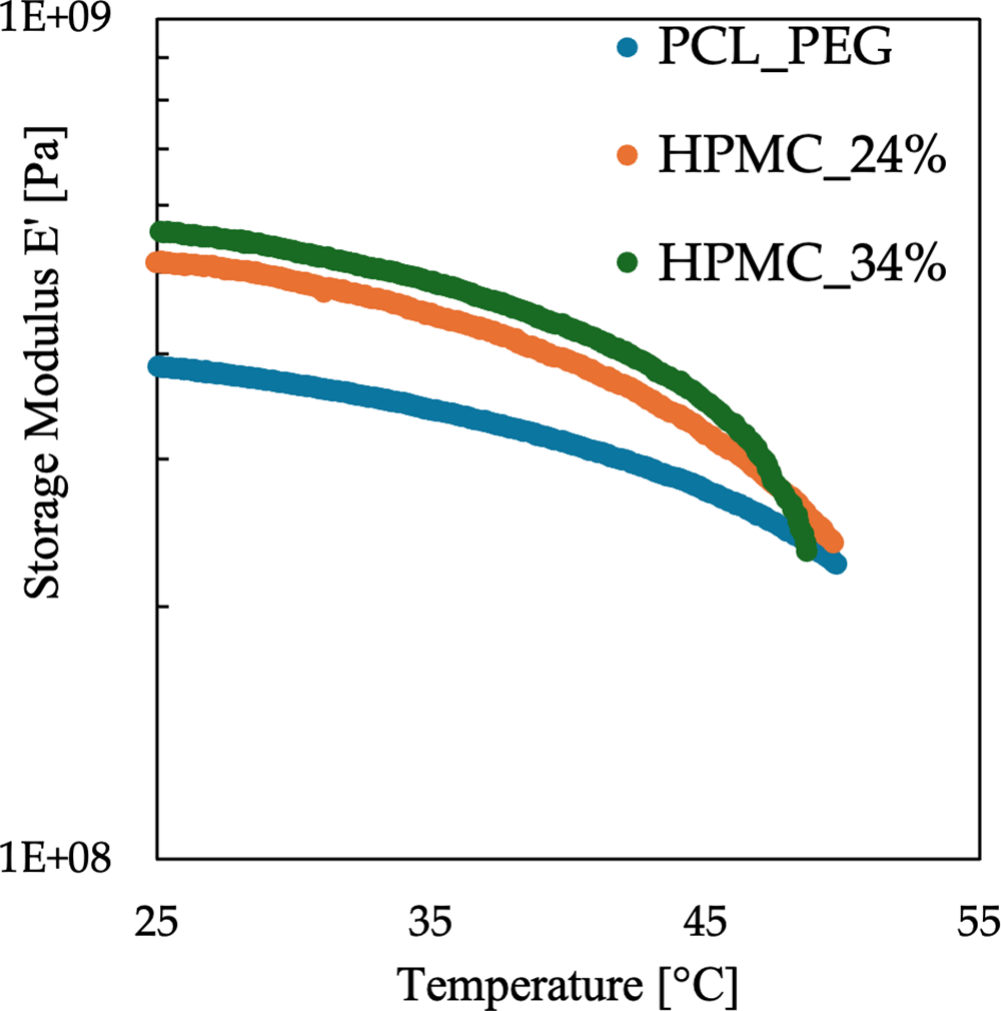
Storage modulus (E′) for blends containing increasing amounts of HPMC

#### 3.1.6 Capsule Production and MB Release Analysis

The capsules were produced by injection molding using the blends prepared by melt compounding with several percentages of HPMC. Figure 5 shows the capsule obtained by injection molding.

**Figure 5:**
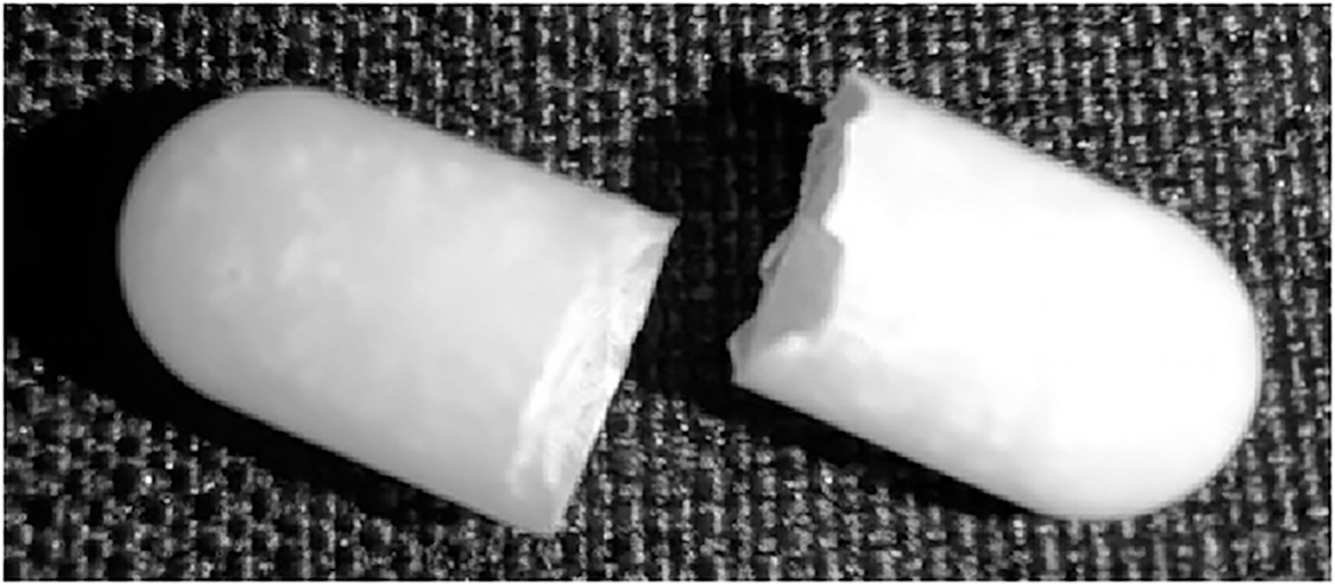
Capsule obtained by injection molding.

Once proper injection molding conditions were assessed, the capsules were assembled and methylene blue was introduced inside the capsules to analyze its release from the polymer matrix.

Figure 6 shows the release profile of methylene blue, adopted to prove the efficiency of the polymeric blends for the proposed application. It has to be noticed that the pH of the release medium changed over time to simulate the release of a drug in the colon: pH is kept at 2.5 until 1.5 h, after that it is increased up to 5 until 3.5 h, finally it is increased up to 6.8 for the remaining time. Figure 6a shows that the amount of methylene blue (MB) released from the PCL/PEG capsule is negligible until 48 h. This result may be attributed to the PCL’s slow degradation kinetics,^38^ which hinder the release of MB from the matrix. The presence of HPMC in the blends significantly modifies the release profile: the higher the HPMC content, the faster the MB release is.

**Figure 6:**
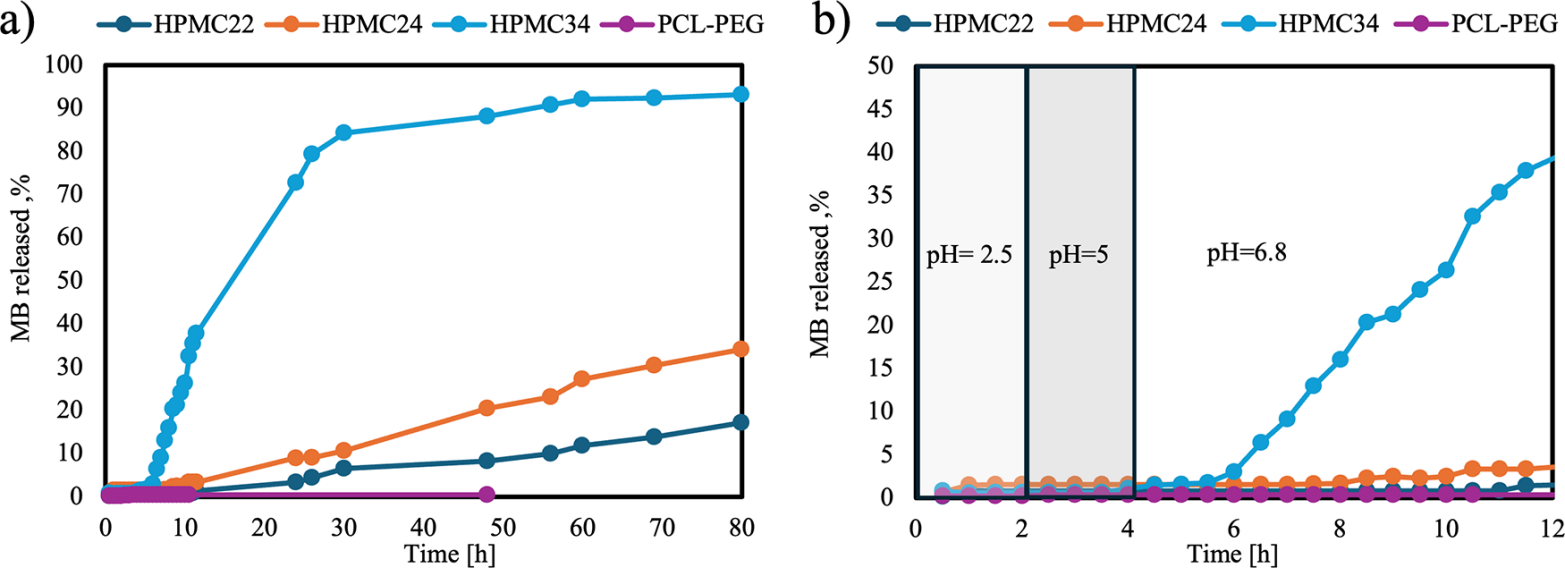
MB release test in the simulated gastrointestinal tract: entire pattern (a) and detail of the first 12 h (b).

It is possible to note that the amount of MB released from the capsules obtained using a percentage of HPMC equal to 22 and 24% is low in the first 30 h (7 and 11%, respectively). In contrast, the release from the capsules containing 34% of HPMC is faster: 84% of MB is released in the same time range.

Figure 6b shows an enlarged view of the release profiles during the first 12 h. In all cases, the MB release exhibits a latency time, a time range during which the release from the capsule is negligible. Capsules containing 22 and 24% HPMC show a latency of about 10–12 h. In contrast, capsules with 34% HPMC content exhibited a shorter latency of around 6 h. It is possible to deduce that, regardless of the HPMC percentage, release occurs only under basic pH conditions.

Comparing this result with other reports in the literature, when only PCL is in a capsule, the release did not occur before 200 h. However, when PEG is added, the release becomes slightly faster; in fact, Liparoti et al. in a previous work reported that varying the percentage of PEG, the release of a model compound starts after 20 h, and the time needed to reach the plateau is closely related to the PEG percentage, but the obtained release is not pH sensitive as in the present work.^39^

It is important to note that the patient’s health considerably influences gastrointestinal (GI) motility.^40^ Indeed, total GI transit time is significantly longer in individuals with severe disorders such as ulcerative colitis compared to healthy subjects. Haase et al.^41^ compared total GI transit time in 20 patients with severe ulcerative colitis to that of 20 healthy volunteers. They observed a transit time of 9.9–102.7 h (median value of 44.5 h) in patients, compared to 9.6 - 56.4 h (median value of 27.6 h) in healthy subjects.

The capsule formulated with the highest HPMC content is well-suited for patients with ulcerative colitis: the capsule with a percentage of HPMC equal to 34% released 73% of MB within the first 24 h (the complete release is obtained in 80 h).

Figure S5 shows the difference in the MB release rates due to the presence of HPMC. The images were obtained over several days, starting at the test’s initiation, at 1, 3, 7, and 15 days. It can be observed that the blue color of the solutions with capsules containing the lower values of HPMC is light and never becomes dark, even after 15 days. The capsule with the highest HPMC concentration seems to release the most significant amount of the MB in a time interval of about 3 days.

Before and after the release tests, the capsules were morphologically characterized by scanning electron microscopy. Figure 7 shows SEM micrographs of the PCL/PEG/HPMC capsule with a 34% HPMC content; the inner surface of the capsule is shown before (left) and after (right) the release test. The pores on the surface of the capsule, before the release test, are small with an average diameter of 10.6 μm. After the release test, the surface of the capsule exhibits an increased, number of pores with enlarged diameter (the average diameter is 19.2 μm).

**Figure 7:**
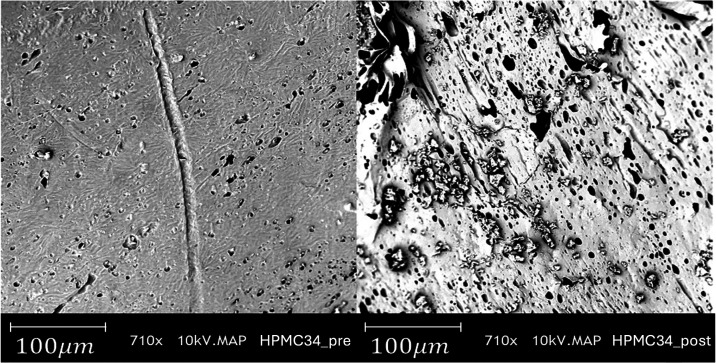
SEM micrographs of the inner surface of the PCL/PEG/HPMC capsule with a 34% HPMC content before (left) and after (right) the release test.

Analyzing the external surface of the same capsule (see Figure 8), it can be observed that the pores formed during the release test are, for the most part, interconnected, with the presence of inner pores having smaller diameters. It can be assumed that the polymer partially solubilizes in PBS at pH = 6.8 and moves away from the matrix, leaving a pore structure that enables the release medium to enter and solubilize MB. This assumption is confirmed by the decrease in peak intensity around 1100–1000 cm^–1^ region, characteristic of HPMC (see Figure S6), observed via ATR-FTIR analyses of capsules after the release test.^34,42^ Instead, the peak at 1645 cm^–1^ is consistent with the methylene blue^43^ remaining superficially absorbed on the capsule after the release.

**Figure 8:**
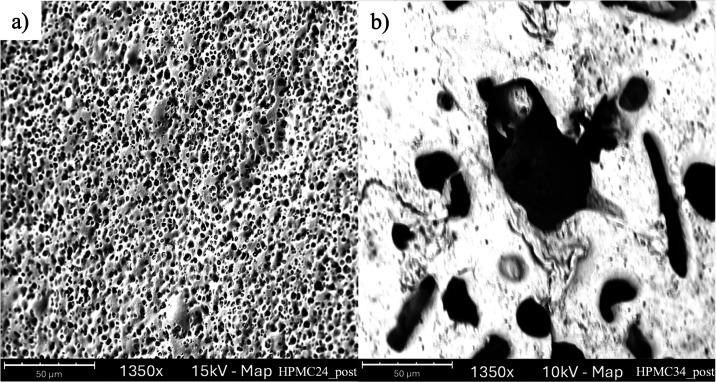
SEM micrographs with the details of the external surface of the capsule after the release test. (a) 24% HPMC; (b) 34% HPMC.

Moreover, it can be observed that the mean diameter and the pore density are strictly related to the amount of HPMC present in the polymeric blend.

Figure 8a shows the surface of the capsule containing 24% HPMC after the release: numerous small pores are visible. In Figure 8b, the surface of the capsule with 34% HPMC after release is shown; in this case, fewer pores with larger diameters are observed. Based on image analysis, the mean pore diameter was estimated to be 4.5 ± 1 μm in the case of 24% HPMC and 32.7 ± 9 μm in the case of 34% HPMC. A higher HPMC content results in larger pores, which may enable a faster release.

### 3.2 Modeling

The analysis of MB release from the capsule suggests that MB is released only after the release medium enters the capsule and solubilizes the MB. Once MB is solubilized in the solution, it can be released into the surrounding medium. Since the MB content within the capsule is well above the saturation threshold (43 g/L;^44^), the release driving force (i.e., the concentration difference between the MB inside and outside the capsule) mainly depends on the accumulation of MB in the release medium. As the concentration of MB inside the capsule drops below the saturation threshold, the driving force diminishes (also due to the MB increasing concentration in the release medium), and the release profile approaches a plateau.

In the literature, it has been reported that most release profiles from porous degradable matrices are governed by the interaction of three main phenomena: polymer erosion, drug diffusion, and the pore structure.^2^ Polymer erosion is generally associated with degradation. PCL shows slow degradation kinetics, with a characteristic time significantly longer than the release time considered in this work. In contrast, HPMC shows high water solubility at pH values above 6.5, which implies an increase in the pore density and size as the release proceeds. Moreover, the initial stage of the release test is conducted under acidic conditions, where HPMC solubility is negligible. Therefore, a latency period must be taken into account.

The model developed to describe MB release through the polymeric matrix accounts for a stage during which the release medium enters the matrix, as given in eq 3.

3 $$W_{a}=SK_{a}\left(c_{a\infty }-c_{\mathrm{ai}}\right)$$

Where *W*_a_ represents the moles of release medium (assumed to have the properties of water) entering the capsule during time (mol/s); *c*_a*∞*_ is the concentration of the release medium in the environment surrounding the capsule; *c*_ai_ is the concentration of the release medium inside the capsule, which increases over time until the capsule volume is filled; *K*_a_ is the mass transfer coefficient (m/s), and *S* is the total capsule surface (m^2^).

*K*_a_ depends on pH; it is assumed to be zero under acidic conditions (pH < 6). Once the release medium has entered the capsule, MB release into the external medium begins. This is described by the following equation

4 $$W_{M}={\mathrm{SK}}_{M}\left(c_{\mathrm{MBi}}-c_{\mathrm{MB}\infty }\right)$$

Where *W*_M_ represents the moles of MB released into the medium during time (mol/s); *c*_MBi_ is the MB concentration inside the capsule. Such a concentration is equal to the solubility threshold in the early stage of the release test; thereafter, it begins to decrease. *c*_MB*∞*_ is the MB concentration in the release medium, which changes over time, according to eq 5 (where *V*_r_ is the volume of the release medium)

5 $$\frac{dc_{\mathrm{MB}\infty }}{\mathrm{dt}}=\frac{W_{M}}{V_{r}}$$

*K*_M_ is the mass transfer coefficient in m/s. This last parameter was taken dependent on the concentration of water that enters the capsule, according to a linear relationship

6 $$K_{M}=k_{M0}c_{\mathrm{ai}}\left(t\right)$$

Where *k*_M0_ is a constant mass transfer coefficient given in Table 4, which also lists the parameter *K*_a_.

**Table 4:** Values of the Mass Transfer Coefficients for the Blends

	HPMC 22%	HPMC 24%	HPMC 34%
*K*_a_ [m/s]	2.95 × 10^–9^	3.44 × 10^–9^	3.93 × 10^–9^
*k*_M0_ [m^4^/(mol s)]	2.75 × 10^–14^	6.39 × 10^–14^	9.43 × 10^–13^

Both *K*_a_ and *k*_M0_ seem to depend on the content of HPMC, in particular, they increase with the percentage of HPMC. This behavior is consistent with the expectations since a more efficient mass transfer is obtained when the pores form in the polymeric matrix. In the first step, the release medium enters the capsule and solubilizes the compound; in the second step, the solubilized compound diffuses out of the polymer matrix. This model accounts for pH-dependent release through a suitable mass transfer coefficient associated with the first step, which is assumed to be zero at pH values lower than 6.8 and to take a constant value at higher pH values, depending on the HPMC content. The mass transfer coefficient for the second step is assumed to vary linearly with the amount of release medium that enters the capsule. Both mass transfer coefficients increase with higher HPMC content in the polymer blend, consistent with faster erosion of the polymer matrix.

Figure 9 shows a comparison between the experimental release profiles and those predicted by adopting the model proposed above. The release of MB predicted by the model is consistent with the experimental profile. The main features of the releases are well captured by the model: a latency time is predicted, and the decrease in the release rate is also predicted once the concentration of MB inside the capsule becomes smaller than the threshold value for solubility.

**Figure 9:**
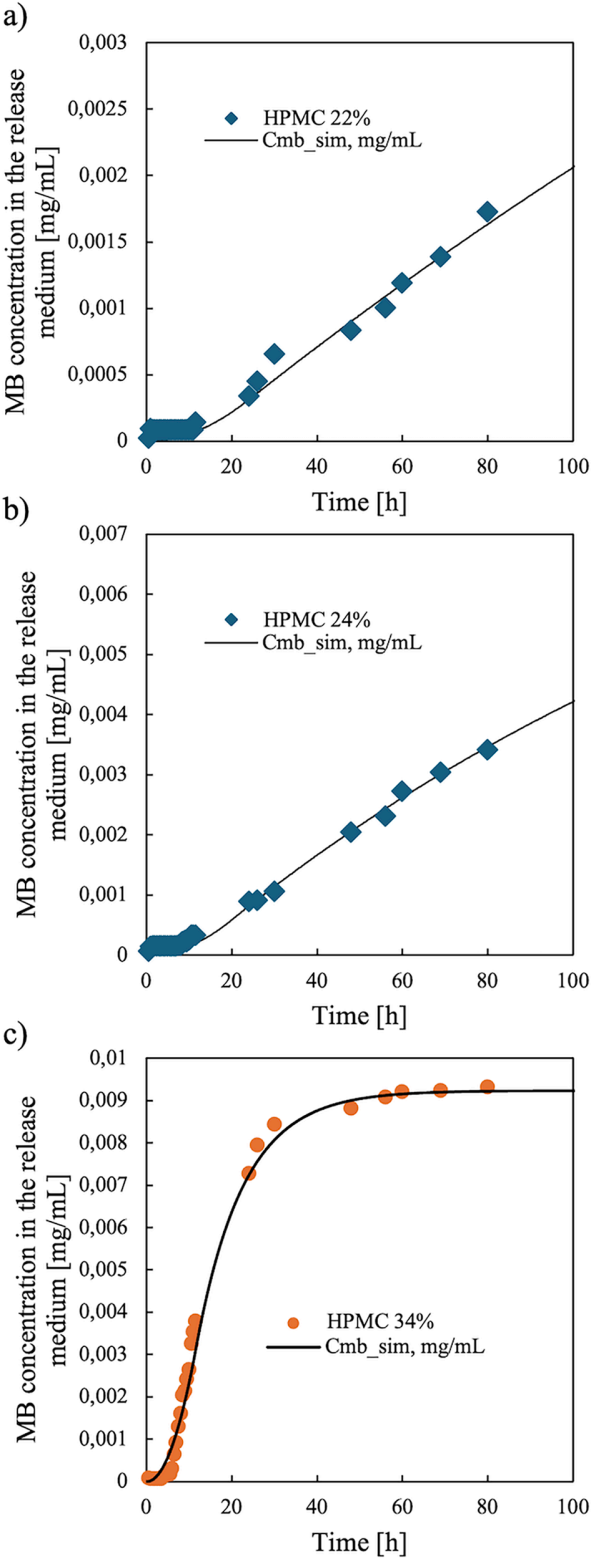
Release profiles evaluated applying the proposed model. Several HPMC percentages were used: (a) 22%, (b) 24%, and (c) 34%.

## 4 Conclusions

In conclusion, this study investigated a custom-made polymeric blend consisting of three different polymers: polyethylene glycol (PEG), polycaprolactone (PCL), and hydroxypropyl methylcellulose (HPMC), optimized for the manufacturing of capsules suitable for colon-targeted drug delivery using an injection molding process.

The work aimed to modulate drug release rate using pH-responsive dissolution polymers. Indeed, the *in vitro* release tests, performed at pH 2.5, 5, and 6.8 to simulate transit through the gastrointestinal tract, showed no release of the compound under gastric or intestinal conditions, and a controlled, sustained release at colonic pH. Furthermore, the release rate of the model compound was found to depend on the HPMC content in the blend. Specifically, for the blend containing 34% HPMC, about 73% of the compound was released within 24 h.

Therefore, this work demonstrates for the first time the formulation of a polymeric blend using these three polymers that exhibits both pH-sensitive responses and time-dependent release, key characteristics for colon-targeted drug delivery systems. This will enable the use of capsules made by injection molding for the drug release, minimizing the adverse effects in the gastrointestinal tract.

A release model was introduced for describing the two-step release of the model compound from the capsule.

## Supplementary Material



## Notes

TGA curves (Figures S1 and S2), DSC thermograms (Figure S3), and complex viscosity measurements (Figure S4) for pure polymers and blends. Figure S5 shows photos of the release medium taken during the release time, and Figure S6 shows the FTIR of the capsule after the release test (PDF)
