# Supplementary material for: Polycaprolactone/Polyethylene Glycol/Hydroxypropylmethylcellulose Blends: Tailoring Thermomechanical and Rheological Properties for Injection-Molded Capsules for Colon-Targeted Delivery Applications
Source: ACS Appl Bio Mater. 2025 Dec 8;9(1):96–105. doi: 10.1021/acsabm.5c01503 (PMC12776571; doi:10.1021/acsabm.5c01503)
Supplement: Supplementary file 1 [file mt5c01503_si_001.pdf]

Supporting Information

Polycaprolactone/polyethylene glycol/Hydroxypropylmethylcellulose blends: Tailoring thermomechanical and rheological properties for injection-molded capsules for colon-targeted delivery applications

Stefania Mottola, Sara Liparoti*, Andrea Miranda, Iolanda De Marco
Department of Industrial Engineering (DIIn), University of Salerno, Via Giovanni Paolo II, 132, Fisciano, SA 84084, Italy

* sliparoti@unisa.it

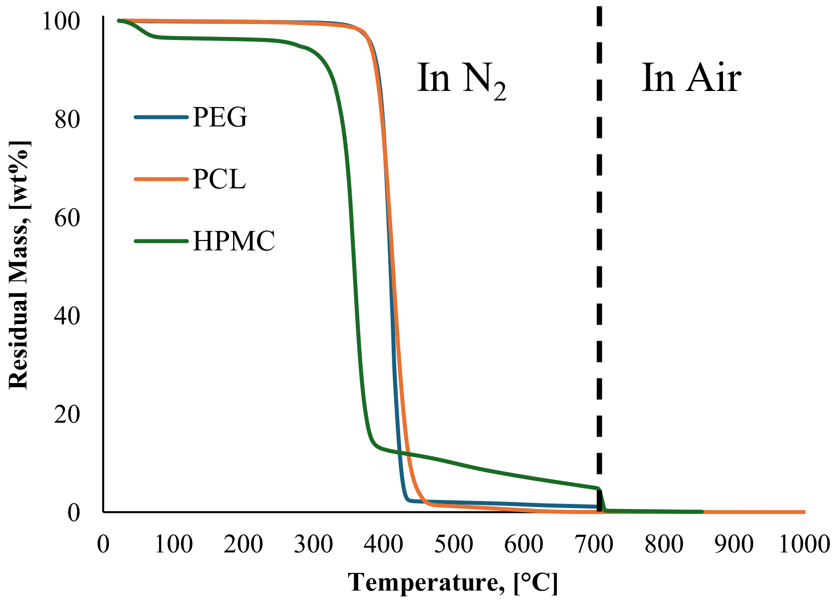

Figure S1. TGA curves of pure polymers. Pure HPMC curve shows the thermal degradation at temperatures in the range 310-389 °C. Both PCL and PEG degraded at higher temperatures (in the range 380-450 °C).

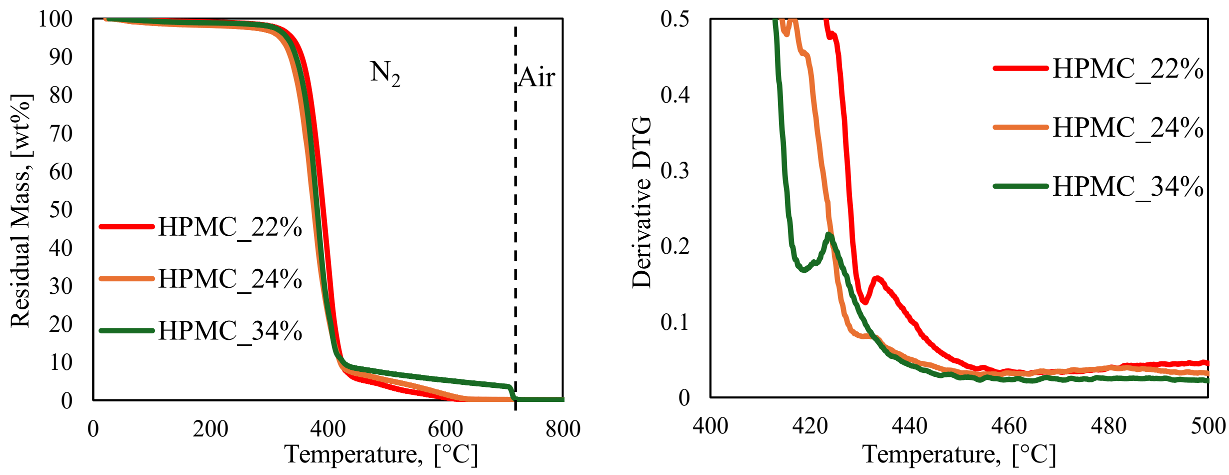

Figure S2.1. TGA curves of blends of PCL, PEG and HMPC; Figure S2.2.Detail of DTG curves of blends.

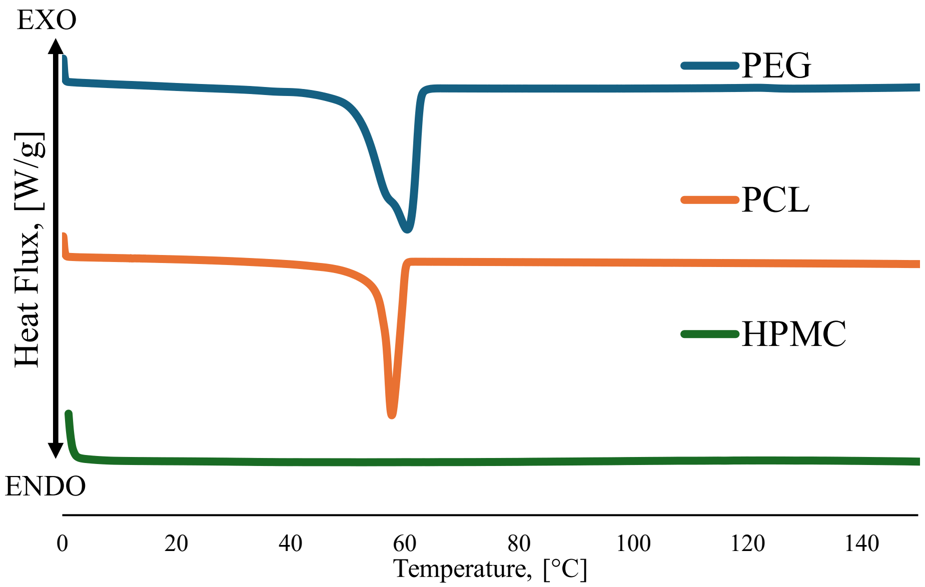

Figure S3. DSC patterns of pure polymers.

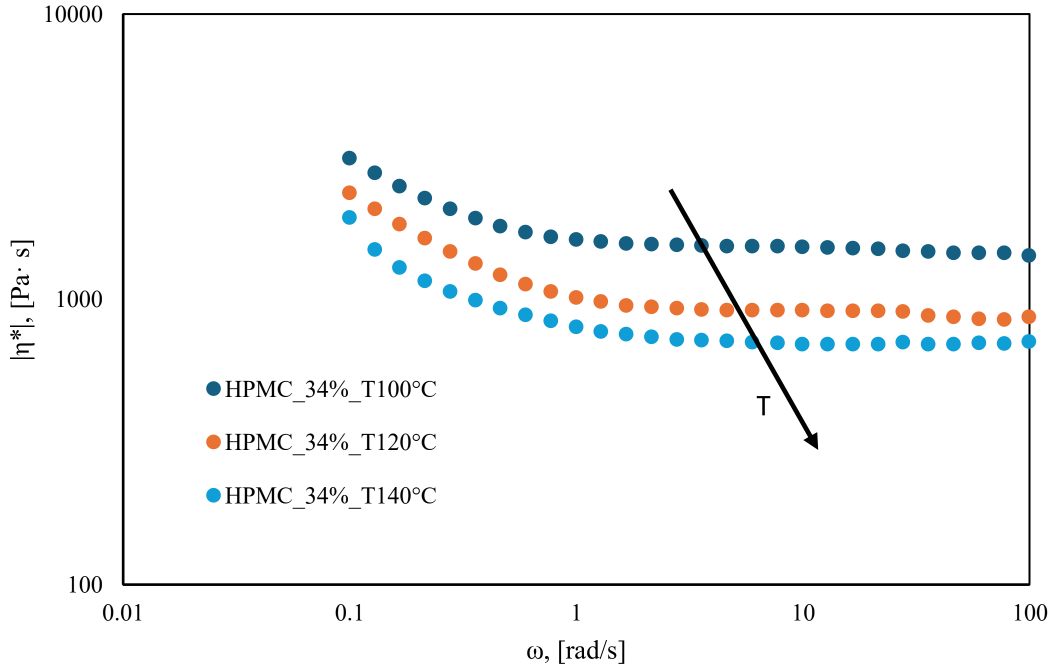

Figure S4. Complex viscosity of the PCL/PEG/HPMC blend with 34%wt of HPMC at different temperature.

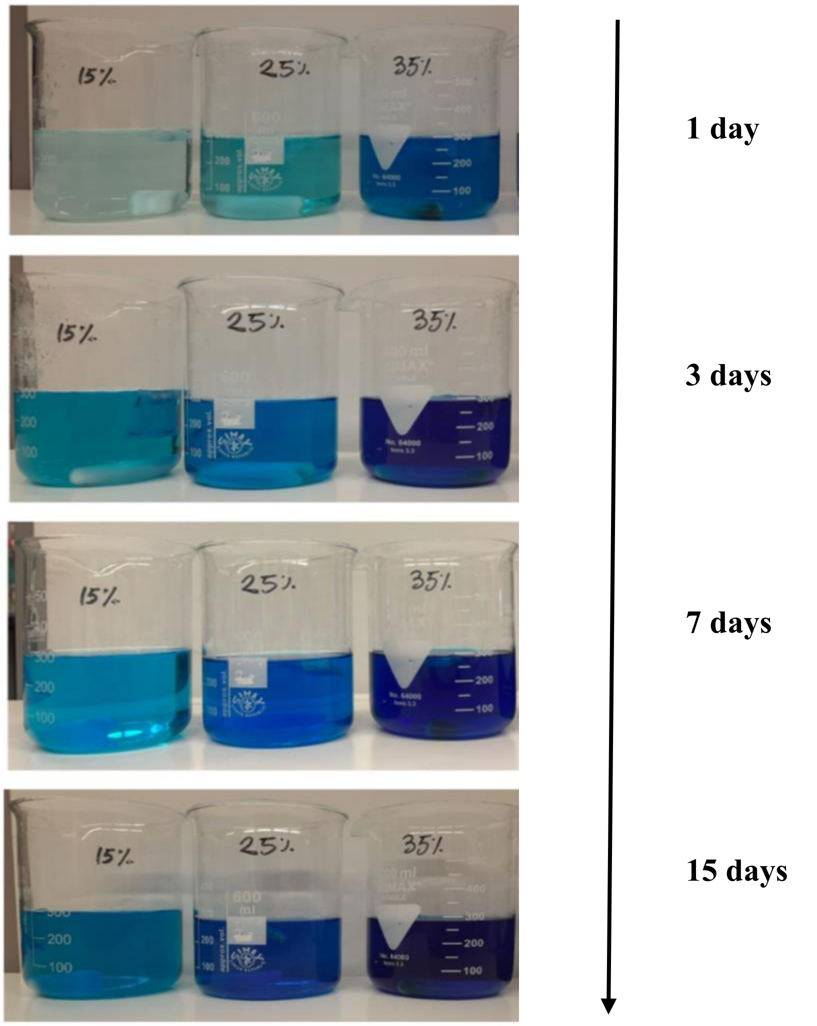

Figure S5. Photos of the release medium at pH = 6.8 after 1, 3, 7, and 15 days.

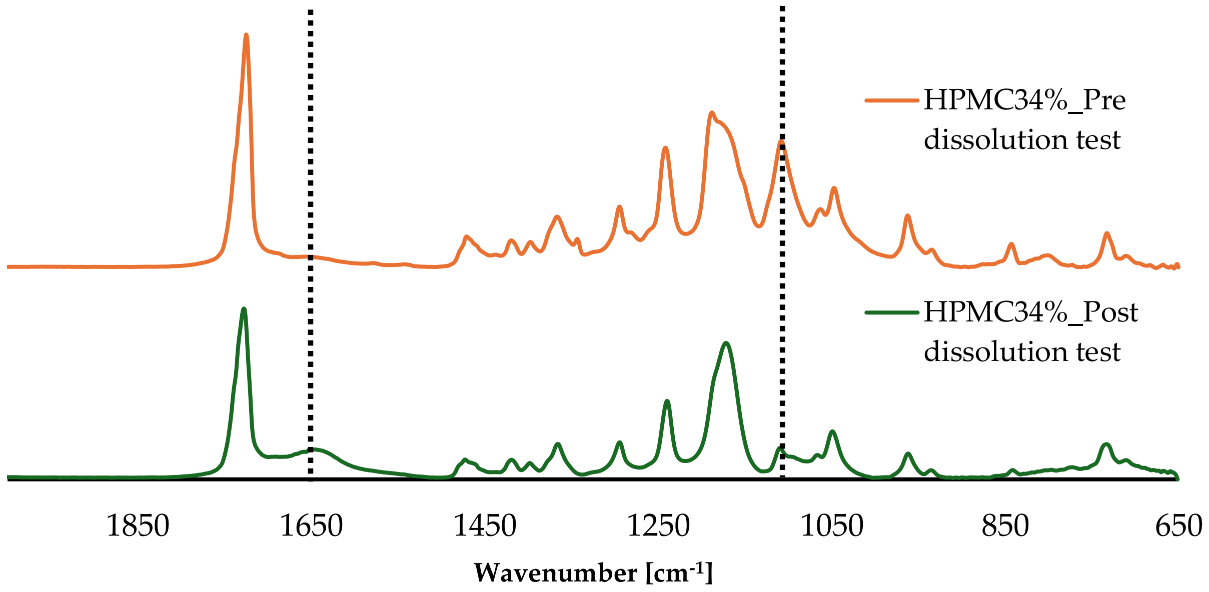

Figure S6. ATR-FTIR of capsule at 35% HPMC before and after the dissolution test, showing the change in HPMC characteristic peak.
